# A Multistate Analysis of Prosthetic and Orthotic Coverage Clarification: Projected Positive Return on Investment and Net Fiscal Benefit

**DOI:** 10.3390/bioengineering13070775

**Published:** 2026-07-03

**Authors:** Shaneis Morse, Prateek Grover, Jeff Cain

**Affiliations:** 1 Upstream Informatics LLC, Lakewood, CO 80215, USA; 2 Department of Physical Medicine and Rehabilitation, Penn State, College of Medicine, Penn State Health Milton S. Hershey Medical Center, Hershey, PA 17033, USA; pgrover@pennstatehealth.psu.edu; 3 Department of Family Medicine, School of Medicine, Colorado University, Anschutz Medical Campus, Aurora, CO 80045, USA; jeffery.cain@cuanschutz.edu

**Keywords:** orthotic devices, prosthesis, artificial limbs, cost benefit analysis, return on investment, preventive health

## Abstract

Background. Orthotic and prosthetic devices for general-use and activity-specific function can provide critical preventive health benefits for individuals with limb loss, limb difference, and mobility impairments, and yet coverage remains inconsistent across U.S. states. Objective. To evaluate the fiscal impact of clarifying insurance coverage for orthotic and prosthetic devices across 23 states lacking comprehensive coverage. Methods. A cost consequence analysis was conducted using data from the U.S. Census Bureau, Kaiser Family Foundation, Government Accountability Office, and a recent actuarial analysis informing baseline cost, coverage, and prevalence assumptions. Per-member-per-month (PMPM) cost increases were compared against device enabled preventive health savings to estimate net fiscal impact. Sensitivity analyses modeled three scenarios based upon a combination of uptake (% eligible individuals accessing device) and physical activity equivalent annual cost saving, respectively: conservative (25% uptake, $1000), moderate (50% uptake, $2500), and high-impact (75% uptake, $5000). Return on investment (ROI) was calculated for the moderate scenario as the ratio of annual savings to implementation cost. Results. Under the assumptions of the moderate scenario, projected ROI remained positive across all states, ranging from approximately 1.5× in Florida to over 114× in Vermont, with 78% of states (18 of 23 states) demonstrating returns greater than 4×. Moderate scenario annual net savings ranged from approximately $10.8 million in Vermont to $437.0 million in California, with substantial projected savings also observed in Florida ($235.5 million), New York ($225.2 million), and Virginia ($143.8 million). PMPM cost increases for 70% of states range between $0.03 and $0.43, with all modeled states remaining below $1.46. Discussion. In our healthcare system dominated by high-cost and reactive care, the ROI obtained by this cost-consequence analysis (CCA) using evidence-based assumptions supports orthotic and prosthetic coverage clarification as preventive interventions to restore function.

## 1. Introduction

While the United States (U.S.) leads global healthcare spending, investing approximately $14.7 K per capita annually [1], life expectancy remains among the lowest among comparable high-income industrialized nations [2]. U.S. life expectancy declined by 2.7 years between 2019 and 2021, marking the largest two-year reduction observed since 1921–1923 [3]. Despite substantial healthcare expenditures, preventable chronic disease continues to contribute significantly to poor population health outcomes and reduced longevity in the United States [4]. Preventive healthcare strategies that improve physical activity and functional mobility have therefore become increasingly important in efforts to reduce long-term healthcare expenditures and improve population health.

The American Heart Association and related cardiovascular health research equate meeting recommended physical activity guidelines (approximately 30 min of moderate activity five days per week) with up to $2500 savings in annual healthcare expenditures compared with inactive individuals, particularly through reductions in cardiovascular-related costs [5,6]. Physical inactivity is additionally recognized by the American Heart Association and World Health Organization as a major modifiable risk factor for cardiovascular disease, contributing substantially to ischemic heart disease, diabetes, and premature mortality [7]. For individuals with limb loss, limb difference, and mobility impairments, barriers to basic standing, transferring, walking, and running limit participation in a healthy lifestyle, increasing long-term cardiovascular, musculoskeletal, and mental health risk. Objective activity-monitoring studies have demonstrated that community-dwelling lower-limb prosthesis users commonly achieve approximately 4000–5000 steps per day, with 5000 daily steps identified as a threshold for a non-sedentary lifestyle, supporting the role of prosthetic mobility in facilitating meaningful physical activity levels [8]. Accordingly, access to orthotic devices and prosthetic devices for both daily functional mobility (general-use) and for specific activities such as showering and swimming, as well as higher levels of physical activity (activity-specific) represent an important form of preventive healthcare with the potential to reduce future chronic disease burden and to reduce associated healthcare expenditures.

Prior studies have demonstrated the cost-effectiveness of orthotic and prosthetic devices [9,10]. However, many states continue to lack comprehensive insurance mandates for these services, which has resulted in insurance companies creating barriers that increase costs, limit access and contribute to poorer health outcomes for amputees. Recent U.S. Government Accountability Office (GAO) analysis reported average prosthetic costs exceeding $13,000 per device, creating a substantial financial burden for individuals responsible for coinsurance or cost-sharing obligations [11]. The GAO additionally identified important equity concerns, including disproportionate impact among Black individuals, elevated mortality rates compared with non-limb-loss populations, and high rates of subsequent amputations [11].

A central policy consideration surrounding orthotic and prosthetic legislation is cost defrayal under the Affordable Care Act (ACA). Under current federal policy, states are responsible for covering the cost of mandated benefits that exceed Essential Health Benefit (EHB) benchmark requirements [12]. However, substantial legal and regulatory grounding supports the position that orthotic and prosthetic services are already encompassed within existing rehabilitative and habilitative EHB categories required for coverage by the ACA. Accordingly, this study evaluates these legislative efforts through the lens of coverage clarification rather than entirely new benefit expansion, while recognizing that interpretations of Essential Health Benefit (EHB) requirements and associated defrayal obligations may vary across jurisdictions and regulatory environments.

The orthotic and prosthetic (O&P) community has consistently argued that activity-specific and custom-fit prosthetic and orthotic devices represent a continuation of existing covered rehabilitative services rather than a novel category of benefit expansion [13]. This interpretation was further reinforced in the 2024 Centers for Medicare and Medicaid Services (CMS) Final Rule establishing the 2025 Benefit and Payment Parameters, which clarified that states may define the scope of benefits within existing EHB categories without necessarily triggering additional state defrayal obligations [14,15]. Consequently, some states may view comprehensive orthotic and prosthetic coverage as consistent with existing EHB frameworks, potentially improving access while limiting additional fiscal liability. However, the applicability of defrayal requirements remains subject to state-specific interpretation and future regulatory guidance.

States with active legislation on clarifying orthotic and prosthetic coverage include Alabama, Alaska, Arizona, California, Delaware, Florida, Georgia, Hawaii, Indiana, Louisiana, Michigan, Nebraska, New York, North Carolina, Oklahoma, Rhode Island, Texas, Utah, Vermont, Virginia, Washington, and Wisconsin. States that have existing general-use device coverage mandates currently pursuing clarification of activity-specific prosthetic and orthotic coverage include California, Delaware, Georgia, Indiana, Louisiana, Missouri, Rhode Island, Texas, Utah, Vermont, Virginia, and Washington [16].

Using multistate cost consequence and return on investment (ROI) modeling, this study evaluates the projected fiscal impact of clarifying commercial and non-group insurance coverage for orthotic and prosthetic devices across these 23 U.S. states.

## 2. Materials and Methods

### 2.1. Study Design

The study was designed as a cost consequence analysis (CCA) within a policy review framework and conducted as a policy simulation using evidence-based assumptions to estimate the fiscal impact of clarified orthotic and prosthetic coverage. All calculations were performed using Microsoft Excel (Microsoft Corporation, Redmond, WA, USA).

### 2.2. Sample Size

Using a population-based modeling approach, the study incorporated statewide non-group and employer-sponsored insurance enrollees across 23 states. This is consistent with the original Minnesota actuarial study framework, which based fiscal estimates on gross expenditures across the total non-public insured population rather than only mandate-applicable fully insured plans.

### 2.3. Duration

The data reflects legislative efforts from 2023 to 2026.

### 2.4. Data Sources

Demographic and insurance coverage data were obtained from the U.S. Census Bureau (2025) [17] and the Kaiser Family Foundation (2025) [18]. Actuarial estimates and cost modeling from the Minnesota Department of Commerce (2024) [19] informed per-member-per-month (PMPM) cost impact in other states. Utilization and cost data from the U.S. Government Accountability Office (GAO) Limb Loss Study (2024) [11] were used to derive prevalence and cost of prosthetic and orthotic device use. Healthcare cost saving projections were informed by research led by Javier Valero-Elizondo, MD, MPH (Baptist Health South Florida) and colleagues with findings published in the Journal of the American Heart Association (JAHA) [5]. Legislative tracking and policy analysis data were included from a prior Fiscal and Social Impact Report [16].

### 2.5. Baseline Model Data Analysis and Assumptions

Using the Minnesota Department of Commerce evaluation report [19] as a baseline, costs were estimated for the 23 modeled states. This approach follows previously published multistate cost consequence analyses [20,21]. PMPM impacts are presented as ranges to reflect uncertainty associated with incomplete state-level claim data and variation in insurance market characteristics across states.

Additionally, Minnesota was selected because it represents one of the few publicly available, comprehensive actuarial evaluations of orthotic and prosthetic coverage incorporating device utilization, claim-based expenditure estimates, covered population projections, and PMPM fiscal impacts. Comparable actuarial analyses were not uniformly available across the 23 modeled states, precluding development of a pooled multistate actuarial baseline. Accordingly, the Minnesota report was utilized as a transparent and replicable starting point for policy modeling. The present analysis does not assume Minnesota is fully representative of all states but rather provides a standardized benchmark from which state-specific population, insurance coverage, disability prevalence, and utilization adjustments could be applied.

This study incorporates publicly available actuarial reports along with modeling assumptions as foundational methodological inputs. Hence, the analysis should be interpreted as a policy and health economics modeling study rather than a formal actuarial certification or actuarial opinion.

#### 2.5.1. Minnesota Per-Device Costs

The first year of coverage for orthotics and prosthetics in MN cost $116,395,832 with a PMPM cost increase of $0.39. With a total of 106,296 devices, per device cost was then calculated as a PMPM divided by the number of devices (Table 1). Orthotics and prosthetic per-device cost were then calculated using respective % expenditure, PMPM cost, and Number of Devices.

#### 2.5.2. Per-State Insured and Disability Rate Differences from MN

For MN, the state insurance coverage (Kaiser Family Foundation 2025) [18] was 61.6%, and the disability rate (U.S. Census 2025) [17] was 7.7% (Table 2). PMPM variance impacts were estimated by multiplying state PMPM estimates by the absolute percentage-point deviation from the Minnesota baseline, expressed as a decimal.

Because both insured population composition and disability burden influence utilization, access, and fiscal exposure, these Minnesota-derived actuarial estimates provide an interpretive framework for evaluating results between heterogeneous state populations.

#### 2.5.3. State Non-Medicare and Relevant Population Estimates

State modeled population estimates were calculated by multiplying the total state population by the percentage of individuals aged 6–64 (Table 2). This age range was selected to approximate the commercially insured population most applicable to the modeled orthotic and prosthetic coverage legislation. Adults aged ≥65 were excluded due to substantial Medicare enrollment, while children under age 6 were excluded because of pediatric prosthetic utilization patterns, growth-related replacement frequency, and coverage dynamics, which differ substantially from adult and adolescent populations.

Importantly, individuals aged 6–18 were retained in the modeled population despite potential overlap with Medicaid enrollment, recognizing that a subset of children in this age group remain.

Covered through commercial or employer-sponsored insurance. Because consistent state-level data distinguishing commercial and Medicaid enrollment were unavailable across all modeled states, this population was retained in the analysis.

#### 2.5.4. Minnesota Device Utilization for 2025

Annual orthotic utilization (1.47%) and annual prosthetic utilization (0.37%) for MN from the 2022 U.S. Census were applied to the 2025 population.

#### 2.5.5. State Prosthetic Utilization, Costs, and PMPM Increase

State general-use prosthetic utilization was calculated by multiplying the state population with the Minnesota prosthetic utilization (0.37%). Activity-specific prosthetic utilization was subsequently estimated by adding 30% to the general-use prosthetic utilization, derived from prior actuarial assumptions reported in a 2025 Maryland orthotic and prosthetic coverage analysis [22]. General-use and Activity-specific prosthetic utilization values were multiplied by per-device prosthetic cost to derive State Prosthetic PMPM costs (Table 3).

#### 2.5.6. State Orthotic Utilization, Costs, and PMPM Increase

State orthotic utilization was calculated as state-relevant population × MN orthotic utilization (1.47%). State orthotic PMPM was then calculated as state orthotic utilization × per-device orthotic costs (Table 3).

For states proposing expansion of both general-use and activity-specific prosthetic coverage in addition to orthotic coverage, PMPM estimates were calculated by summing general-use prosthetic, activity-specific prosthetic, and orthotic PMPM components (GUP + ASP + O; Table 3). These states included Alabama, Alaska, Arizona, Florida, Hawaii, Michigan, Nebraska, New York, North Carolina, Oklahoma, and Wisconsin.

For states with existing general-use prosthetic coverage mandates, PMPM estimates reflected activity-specific prosthetic and orthotic components only (ASP + O; Table 3). These states included California, Delaware, Georgia, Indiana, Louisiana, Missouri, Rhode Island, Texas, Utah, Vermont, Virginia, and Washington.

Additional variance adjustments (V) were evaluated using differences in insured population composition and disability prevalence relative to the Minnesota baseline. PMPM variance impacts were estimated by applying state-level deviations in total non-public insured populations and disability prevalence to modeled PMPM estimates. Variance adjustments projected to increase PMPM estimates by greater than $0.01 were incorporated into final PMPM calculations, annual cost estimates, net fiscal impact projections, and ROI analyses to improve state-specific fiscal accuracy.

Variance impacts below $0.01 PMPM were not separately incorporated into final state estimates. This threshold was selected as a practical materiality cutoff intended to distinguish minor variation from differences likely to meaningfully influence overall fiscal projections. Because PMPM estimates in this analysis ranged from approximately $0.03 to $1.46, adjustments below $0.01 represented a relatively small proportion of total projected costs. The threshold was not intended to imply statistical insignificance, but rather to simplify interpretation within a policy simulation framework while preserving larger state-specific variance effects.

Final PMPM estimates therefore reflected either GUP + ASP + O + V or ASP + O + V, depending on the structure of proposed state legislation and applicable variance adjustments (Table 3).

#### 2.5.7. Savings Through Physical Activity by O&P Users

Savings from increased physical activity by O&P users with appropriate prosthetic device coverage were calculated using the total O&P utilizing population multiplied by $2500 (Table 4). This value is derived from prior cardiovascular health economics research demonstrating that individuals meeting recommended physical activity levels incur approximately $2500 lower annual healthcare expenditures compared with inactive individuals [5]. While prosthetic provision alone does not guarantee sustained physical activity, evidence suggests that restoration of ambulatory capacity enables meaningful levels of community-based walking among individuals with limb loss. Miller et al. demonstrated that community-dwelling lower-limb prosthesis users commonly achieve approximately 4000–5000 steps per day, with 5000 steps identified as a threshold for a non-sedentary lifestyle [8]. These activity levels generally correspond to approximately 40–60 min of accumulated walking throughout the day. Accordingly, the model assumes that prosthetic access enables participation in health-promoting physical activity at levels reasonably comparable to those associated with the healthcare savings reported by Valero-Elizondo et al. [5], while recognizing that realized savings may vary among individuals.

#### 2.5.8. Annual Cost to All Members

Calculated as number of members × 12 × PMPM. (Table 4).

#### 2.5.9. Net Savings to State

Calculated as the difference of in savings through physical activity by O&P users and annual cost to all members (Table 4).

### 2.6. Sensitivity Analysis

The baseline model assumes that individuals who successfully utilize orthotic and prosthetic devices may achieve healthcare savings associated with increased physical activity and restored mobility, as described by Valero-Elizondo et al. [5]. However, this assumption represents an idealized scenario. In real-world settings, variability exists in both the magnitude of savings achieved per individual and the proportion of individuals who experience these benefits. This variability may be influenced by differences in patient adherence, functional capacity, access to rehabilitation services, and broader social determinants of health.

Sensitivity analysis was therefore employed to test how changes in these assumptions affect overall fiscal outcomes. This approach allows for assessment of whether projected net savings remain stable under more conservative scenarios, thereby strengthening confidence in the model.

Two parameters were varied: (1) annual healthcare savings per individual related to device-enabled physical activity and (2) uptake of activity-related benefit among the device-utilizing population, defined as the proportion of individuals who, after receiving a prosthetic or orthotic device, engage in sustained increases in physical activity sufficient to generate measurable health improvements (e.g., reduced cardiovascular risk or utilization). This parameter captures the behavioral component of device use, recognizing that access alone does not guarantee consistent or effective utilization.

#### 2.6.1. Scenarios

For the conservative scenario, annual savings were reduced to $1000 per individual, representing 40% of the baseline estimate ($1000 ÷ $2500 = 0.40). Uptake was limited to 25% of the utilizing population, reflecting a scenario in which only a subset of individuals both adopt and maintain activity levels necessary to realize downstream health benefits. These adjustments were combined multiplicatively, resulting in a scaling factor of 0.10 (0.40 × 0.25), applied to baseline savings.

For the moderate scenario, baseline savings of $2500 per individual were maintained, while uptake was reduced to 50%, representing partial behavioral adoption across the population, resulting in a scaling factor of 0.50.

For the high-impact scenario, annual savings were increased to $5000 per individual, representing 200% of the baseline estimate ($5000 ÷ $2500 = 2.00), with uptake assumed at 75%, reflecting a scenario of widespread and sustained engagement in physical activity. This resulted in a scaling factor of 1.50.

#### 2.6.2. Adjusted Savings for Each State

Calculated as the product of baseline savings and scenario scaling factor.

#### 2.6.3. Net Cost Savings

Calculated as the difference between adjusted savings and annual cost to all members (Table 5).

This methodology isolates the impact of uncertainty in both behavioral response and intervention effectiveness while preserving the cost structure derived from Minnesota actuarial data. By evaluating outcomes across a range of plausible conditions, the analysis provides a more comprehensive assessment of fiscal risk and potential return, ensuring that conclusions are not dependent on a single set of assumptions.

### 2.7. Return on Investment (ROI)

To further evaluate the fiscal efficiency of expanded prosthetic and orthotic coverage, a return on investment (ROI) metric was calculated for each state.

ROI was defined as the ratio of moderate scenario net savings to state and annual cost to all members (i.e., moderate scenario net savings/annual cost). This scenario assumes that 50% of the estimated maximum potential savings are realized. This approach balances conservative and optimistic assumptions and aligns with prior cost consequence modeling practices.

An ROI greater than 1.0 indicates that projected savings exceed implementation costs, representing a net positive fiscal return. ROI values were calculated for each state to enable cross-state comparison of economic efficiency and to assess scalability across varying population sizes and policy structures. Results are presented in Table 5.

## 3. Results

### 3.1. State-Level PMPM Cost Projections

Across all 23 states analyzed, clarification of orthotic and prosthetic coverage to include orthotic, general-use and activity-specific prosthetic devices was associated with minimal per-member-per-month (PMPM) cost increases and substantial projected annual net savings. Final PMPM increases, including applicable variance adjustments (V), ranged from $0.03 to $1.46, with most states (70%) remaining below $0.43 (Table 3). States with lower projected utilization or smaller covered populations demonstrated PMPM increases closer to $0.03 to $0.30, while larger population states with higher projected utilization, including California, Florida, New York, and Texas, demonstrated comparatively higher PMPM increases.

State-specific prosthetic and orthotic utilization estimates, variance adjustments, cost projections, and PMPM increases are summarized in Table 3.

Comparison of state-level insurance coverage and disability prevalence relative to Minnesota demonstrated modest variation in modeled fiscal impact. Louisiana exhibited the largest deviation in insured population composition (−15% relative to Minnesota), whereas Alabama demonstrated the largest disability prevalence deviation (+3.9%). Application of disability prevalence adjustments produced PMPM variance impacts equal or greater than $0.01 in seven states (Alabama, Delaware, Florida, Indiana, Michigan, North Carolina, and Vermont). Insured population composition adjustments produced PMPM variance impacts greater than $0.01 in fourteen states (Alabama, Arizona, California, Florida, Georgia, Indiana, Louisiana, Michigan, Missouri, New York, North Carolina, Oklahoma, Texas, and Virginia).

### 3.2. Net Fiscal Impact Projections

Despite variation in PMPM estimates, all states demonstrated positive projected net fiscal impact under the baseline annual savings assumption of $2500 per individual (Table 4). Estimated annual net savings ranged from approximately $21.8 million in Vermont to over $1.14 billion in California. Larger states, including Texas ($980.4 million), Florida ($631.0 million), New York ($574.4 million), and North Carolina ($354.6 million), demonstrated substantial projected fiscal impact due to larger covered populations and higher estimated utilization.

Mid-sized states also demonstrated substantial projected net savings, including Michigan ($318.9 million), Virginia ($301.5 million), Washington ($274.1 million), and Arizona ($246.0 million). Smaller states, including Rhode Island ($38.6 million), Delaware ($35.6 million), and Alaska ($26.7 million), demonstrated comparatively lower but consistently positive projected net fiscal impact.

Estimated annual gross savings, annual implementation costs, and net fiscal impact projections by state are summarized in Table 4.

### 3.3. Sensitivity Analysis

Under conservative assumptions, defined by reduced annual savings ($1000 per individual) and limited uptake (25%), most states continued to demonstrate positive projected net savings, although at reduced magnitudes. Annual net savings under this scenario ranged from approximately $2.1 million in Vermont to $17.7 million in Virginia. North Carolina demonstrated near cost neutrality (−$1.4 million), while California (−$129.3 million), Florida (−$81.0 million), Texas (−$69.8 million), and New York (−$54.2 million) demonstrated net costs under conservative assumptions, reflecting sensitivity to lower behavioral uptake and reduced per-individual savings in states with larger implementation costs.

Under moderate assumptions, defined as baseline annual savings with 50% uptake, all states demonstrated strong positive projected net fiscal impact. Net savings ranged from approximately $10.8 million in Vermont to over $437.0 million in California. Larger states, including Texas ($397.0 million), Florida ($235.5 million), New York ($225.2 million), and North Carolina ($156.8 million), maintained substantial projected fiscal benefit.

Under high-impact assumptions, defined as increased annual savings ($5000 per individual) and higher uptake (75%), all states demonstrated substantial positive fiscal outcomes. Net savings ranged from approximately $32.7 million in Vermont to over $1.85 billion in California. Additional high-impact states included Texas ($1.56 billion), Florida ($1.03 billion), and New York ($923.5 million).

Overall, sensitivity analyses demonstrated that while projected savings were influenced by uptake and annual healthcare savings assumptions, all states maintained positive fiscal outcomes under moderate and high-impact scenarios.

### 3.4. Return on Investment (ROI)

ROI remained positive across all states, demonstrating that projected savings from clarified prosthetic and orthotic coverage to include orthotic, general-use and activity-specific prosthetic devices exceeded associated implementation costs in every moderate and high-impact modeled scenario (Table 5). ROI estimates ranged from approximately 1.5× in Florida to over 114× in Vermont, with approximately 78% of states demonstrating returns greater than 4×.

Larger states with higher baseline implementation costs, including California (1.6×), New York (1.8×), and Texas (2.1×), demonstrated more modest but consistently positive returns. In contrast, smaller states, including Vermont (114.4×), Rhode Island (99.1×), and Delaware (83.9×), demonstrated substantially higher ROI values, reflecting lower implementation costs relative to projected downstream healthcare savings.

Mid-sized states demonstrated strong and consistent returns, including Nebraska (25.6×), Louisiana (24.0×), and Utah (22.1×), indicating favorable scalability across diverse population sizes and insurance markets. These findings suggest that even under moderate uptake and savings assumptions, projected savings consistently outweigh expenditures across diverse state insurance environments. ROI estimates by state are summarized in Table 5.

## 4. Discussion

### 4.1. Study Findings and Social Implications

This updated cost-consequence analysis across 23 states reinforces the fiscal and social rationale for clarifying orthotic, general-use prosthetic, and activity-specific prosthetic device coverage as a healthcare mandate for commercial payers. The central finding remains consistent: projected per-member-per-month (PMPM) increases remain minimal, while the downstream savings associated with improved mobility and physical activity are substantial. Even at the upper bound, PMPM impacts remain modest relative to the significant access gains for individuals with mobility impairments. These findings are consistent with prior state-level and single-state actuarial analyses demonstrating favorable cost and outcome profiles associated with expanded orthotic and prosthetic coverage [22].

Importantly, the addition of return on investment (ROI) analysis provides an additional framework for evaluating the potential fiscal implications of coverage clarification. Under moderate assumptions, all states demonstrated a projected positive ROI, indicating that projected savings exceeded implementation costs in all modeled environments. ROI values ranged from approximately 1.5× in Florida to over 114× in Vermont, with approximately 78% of states demonstrating ROI values greater than 4×. These findings indicate that expanded coverage is projected to be cost saving in aggregate and may represent an efficient allocation of healthcare resources across diverse state contexts.

Increased access to mobility devices may contribute to improved functional mobility and greater participation in physical activity, factors that have been associated with reduced risk of obesity, cardiovascular disease, and secondary musculoskeletal complications [23,24]. While the present analysis does not directly evaluate downstream clinical outcomes or healthcare utilization, these established relationships provide a plausible mechanism through which expanded orthotic and prosthetic access could generate long-term health and economic benefits. Consequently, projected savings should be interpreted as estimates derived from evidence-informed assumptions rather than direct measurements of realized health-system impact.

Across the 23-state analysis, the aggregate projected annual net fiscal benefit is substantial, reaching into the billion-dollar range under moderate and high-impact scenarios. High-impact scenarios demonstrate nonlinear scaling of savings in larger states, reflecting population-driven amplification of preventive health benefits. These findings reinforce prior state-level analyses (e.g., Minnesota) demonstrating improved health outcomes alongside reductions in disparities. Collectively, the results suggest that expanding orthotic and prosthetic coverage may generate meaningful economic and health benefits under the modeled scenarios.

### 4.2. Methodological Considerations

This study leverages publicly available data sources, including actuarial analyses, U.S. Census data, and Kaiser Family Foundation estimates, to ensure transparency and replicability. It builds upon prior single-state analyses by applying standardized assumptions across a broader, multistate context. While publicly available actuarial reports and actuarial modeling assumptions inform baseline PMPM estimates and utilization projections, this study should be interpreted as a policy and health economics modeling analysis rather than a formal actuarial certification or actuarial opinion.

A key methodological input is the American Heart Association’s estimate of approximately $2500 in annual healthcare savings associated with regular physical activity. By linking device access to increased functional mobility, the analysis models a plausible pathway through which clinical benefit may translate into economic impact. However, the relationship between coverage expansion, physical activity, and downstream healthcare utilization remains an assumption of the policy model rather than a directly measured outcome. This assumption is supported by objective activity-monitoring studies demonstrating that community-dwelling prosthesis users commonly achieve approximately 4000–5000 daily steps, equivalent to roughly 40–60 min of accumulated ambulation, after restoration of mobility. State-level adjustments, such as population size, insurance coverage, and disability prevalence, allow for more tailored projections while maintaining comparability across states.

The inclusion of ROI as a complementary metric enhances interpretability by contextualizing net savings relative to implementation cost. While total savings quantify aggregate fiscal impact, ROI provides a standardized measure of efficiency, enabling comparison across states with varying population sizes and baseline expenditures. This is particularly important given observed variability in PMPM estimates and total costs across states.

To improve interpretability, the model incorporated a practical PMPM materiality threshold for state-level variance adjustments. While alternative thresholds could modestly influence state-specific projections, the primary findings remained driven by differences substantially larger than the excluded adjustments.

Importantly, findings are consistent with broader federal analyses, including Government Accountability Office (GAO) reports documenting disparities in access to prosthetic and orthotic care. This study extends that evidence base by offering a clear, actionable policy pathway to address those disparities while maintaining fiscal responsibility.

The distinction between coverage clarification and benefit expansion has important policy and fiscal implications under the Affordable Care Act (ACA). Because orthotic and prosthetic services may reasonably be interpreted as existing rehabilitative and habilitative Essential Health Benefits (EHBs) required by the ACA, some states may determine that more comprehensive access standards can be incorporated within existing benefit categories. However, interpretation of EHB requirements and associated defrayal obligations remains subject to state-specific policy decisions and evolving regulatory guidance [12,13,14,15]. Recent CMS guidance further supports state flexibility in defining the scope of benefits within existing EHB categories, strengthening the legal and regulatory feasibility of broader orthotic and prosthetic coverage clarification efforts.

From a policy perspective, the ROI findings provide a useful framework for evaluating the potential fiscal implications of expanded orthotic and prosthetic coverage. Under the assumptions of the moderate scenario, all modeled states demonstrated projected ROI values greater than 1.0, indicating that estimated healthcare savings exceeded projected implementation costs. The moderate scenario was selected as a midpoint between conservative and high-impact assumptions and was intended to represent a plausible policy scenario rather than a prediction of realized performance. Accordingly, ROI estimates should be interpreted as model-based projections rather than forecasts of future financial performance.

Additionally, ROI is inherently sensitive to the relationship between projected savings and implementation costs. In smaller states with relatively low implementation costs, modest changes in assumptions may produce disproportionately large ROI values. Accordingly, exceptionally high ROI estimates should be interpreted as reflecting the mathematical properties of ratio-based measures rather than evidence of guaranteed fiscal returns.

While these findings should be interpreted within the context of the model assumptions, they suggest that clarified orthotic and prosthetic coverage may represent not only a clinically meaningful intervention, but also a potentially cost-effective preventive health strategy. This perspective may be particularly relevant in larger states where implementation costs are greater, yet projected downstream savings continue to offset the associated fiscal investment under the modeled scenarios.

### 4.3. Study Assumptions, Limitations and Future Research

First, this analysis applies intentionally conservative assumptions to avoid overstating fiscal impact. Due to limited access to comprehensive all-payer claim databases (APCDs) across all states, Minnesota’s actuarial data served as the baseline for PMPM estimates, coverage rates, and disability prevalence. Access barriers to APCD data remain practical limitations for multistate analyses [25]. Importantly, when state-specific claim validation data have become available, subsequent actuarial refinement has continued to demonstrate relatively modest PMPM impacts. For example, a revised 2025 Maryland APCD-based orthotic analysis [22] projected PMPM estimates approximately 18% lower increases ($0.13 vs. $0.16 PMPM) than prior broader modeling estimates reported in a methodologically similar 2024 cost consequence analysis (CCA) [20]. This would suggest that more granular claim based validation would further refine and potentially lower projected fiscal exposure while maintaining the overall directionality of low-cost impact findings.

The decision to exclude variance adjustments below $0.01 PMPM represents a modeling assumption rather than a formally validated actuarial standard. Although these adjustments were small relative to the overall PMPM range observed across states, minor differences may accumulate when applied across large insured populations. Consequently, state-specific fiscal estimates should be interpreted as directional projections rather than precise actuarial forecasts. Future analyses using state-specific claim data and actuarial modeling could evaluate the sensitivity of projected costs to alternative variance-adjustment thresholds.

Additionally, state healthcare systems differ substantially with respect to provider availability, rehabilitation access, payer mix, socioeconomic characteristics, utilization patterns, and prosthetic prescribing practices. Although state-specific adjustments for disability prevalence and insurance coverage composition were incorporated, these factors cannot fully capture the complexity of state-level healthcare heterogeneity. Modeled PMPM estimates and fiscal impacts should therefore be read as directional rather than precise state-specific forecasts. Future analyses incorporating state all-payer claim databases, carrier-specific enrollment data, and additional actuarial evaluations could further refine these projections.

The inclusion of individuals aged 6–18 may modestly overestimate the mandate-applicable population because some children within this age range receive Medicaid coverage rather than commercial insurance. However, consistent state-level enrollment data capable of distinguishing coverage sources were not uniformly available across all modeled states. Retaining this population was intended to avoid potential underestimation of utilization and fiscal exposure within commercially insured pediatric populations and reflects a conservative modeling approach.

Second, estimated healthcare savings assume that restored mobility can facilitate meaningful participation in physical activity. Recent accelerometer-based studies demonstrate that community-dwelling prosthesis users commonly achieve approximately 4000–5000 steps per day, corresponding to roughly 40–60 min of daily ambulation. However, device access does not guarantee sustained physical activity, and activity levels will vary according to individual health status, functional capacity, rehabilitation access, and long-term adherence. Furthermore, the analysis does not directly measure the causal impact of orthotic and prosthetic coverage expansion on obesity, cardiovascular disease, musculoskeletal complications, healthcare utilization, or healthcare system capacity. Rather, projected benefits are derived from established evidence linking mobility and physical activity to improved health outcomes. Consequently, healthcare savings should be interpreted as reasonable proxies for the potential benefits of restored mobility rather than guarantees of realized outcomes.

ROI estimates are dependent upon assumptions regarding both behavioral uptake and annual healthcare savings. Although the moderate scenario reduced uptake to 50% of the device-utilizing population, it assumes that individuals who realize benefits achieve healthcare savings consistent with the baseline estimate derived from the physical activity literature. Actual utilization patterns, rehabilitation access, adherence, and health outcomes may vary considerably across individuals and states. Consequently, projected ROI values should be interpreted as illustrative policy estimates rather than actuarial forecasts. This consideration is particularly relevant in smaller states, where relatively low implementation costs may amplify ratio-based ROI calculations and produce unusually large estimated returns.

The sensitivity analysis was intentionally designed to evaluate uncertainty surrounding the two model parameters expected to exert the greatest influence on fiscal outcomes: annual healthcare savings and behavioral uptake. Other sources of uncertainty, including prosthetic replacement frequency, maintenance costs, rehabilitation adherence, provider availability, regional reimbursement variation, insurance market composition, and administrative implementation costs, were not explicitly modeled. Future analyses may benefit from probabilistic approaches such as Monte Carlo simulation to simultaneously evaluate uncertainty across multiple model inputs and further characterize the robustness of projected fiscal outcomes.

Third, certain baseline cost components (e.g., bathing and showering devices) were retained due to insufficient claim-level granularity to reliably isolate and exclude them from the analysis. Finally, fiscal impacts were modeled on an annual basis and therefore do not capture longer-term cost trajectories or cumulative savings associated with sustained mobility, chronic disease prevention, and improved health outcomes over multiple years. As a result, projected savings may underestimate the full long-term economic benefits of expanded orthotic and prosthetic coverage.

Additionally, publicly available insurance enrollment datasets present important limitations when estimating state-specific commercially insured populations potentially subject to mandate applicability. While Kaiser Family Foundation datasets provide broad estimates of employer-sponsored and non-group coverage, they do not consistently differentiate between fully insured commercial plans, ERISA self-funded plans, administrative services only (ASO) arrangements, level-funded products, and other commercial enrollment structures across states. Because state orthotic and prosthetic coverage mandates generally apply only to fully insured plans, broader commercial enrollment estimates were utilized to avoid underestimating potential utilization and fiscal exposure in the absence of more granular state-level data. Future analyses incorporating all-payer claim databases, carrier-level enrollment reporting, or proprietary actuarial datasets could further refine estimates of mandate-applicable populations and associated fiscal impacts [26].

An additional consideration is that state orthotic and prosthetic coverage mandates generally apply only to fully insured commercial plans and do not uniformly apply to ERISA self-funded employer plans. Because publicly available enrollment data do not consistently distinguish between these market segments across all modeled states, the analysis uses broader commercial insurance population estimates as a proxy for potentially affected populations. Consequently, the modeled population may overestimate the number of individuals directly subject to state mandate requirements, which could affect both projected implementation costs and projected savings. However, because both costs and savings scale proportionally with the affected population, the primary findings regarding the relative relationship between projected expenditures and potential downstream benefits are expected to remain directionally informative. Future analyses incorporating carrier-level enrollment data and more precise estimates of mandate-applicable populations could further refine these projections.

The policy analysis presented in this study reflects current federal guidance and publicly available interpretations regarding Essential Health Benefits (EHBs), rehabilitative and habilitative services, and state defrayal obligations under the Affordable Care Act. While recent federal guidance has provided states with additional flexibility in defining benefit scope within existing EHB categories, interpretations may vary across jurisdictions and remain subject to future regulatory, legislative, or judicial developments. Consequently, the discussion of coverage clarification should be interpreted as one potential policy framework rather than a definitive legal determination regarding state defrayal obligations.

Future research should evaluate realized longitudinal outcomes associated with expanded orthotic and prosthetic coverage using state all-payer claim databases and payer-specific utilization data. Additional investigation into sustained physical activity adherence, long-term cardiovascular outcomes, and reductions in secondary musculoskeletal complications would further strengthen understanding of the population-level impact of orthotic and prosthetic access. Prospective post-implementation analyses may additionally help validate and refine future policy and economic modeling approaches.

## 5. Conclusions

Clarified orthotic and prosthetic coverage across 23 modeled U.S. states was associated with low PMPM increases, positive projected net fiscal impact, and favorable ROI estimates under most sensitivity scenarios. Under the assumptions of the modeled scenarios, findings suggest that broader access to mobility-supporting devices may represent a fiscally viable preventive health strategy with potential implications for chronic disease prevention, healthcare system efficiency, and health equity. Future validation using longitudinal claim data and post-implementation evaluations is warranted to assess the extent to which these projected benefits are realized in practice. While future validation using longitudinal claim data is warranted, these results support continued evaluation of orthotic and prosthetic coverage clarification within state insurance policy frameworks.

## Figures and Tables

**Table 1:** Calculation of Minnesota Per Device Per Member Cost Based on Minnesota Actuarial Data.

Device Category	Expenditure, Annual (%)	Expenditure, Annual (USD)	PMPM * (USD)	Number of Devices	Per-Device PMPM Contribution ^1^
Total	100	116,395,832	0.39	106,296	0.0000037
Orthotics	44.7	52,075,495	0.17	84,776	0.00000206
Prosthetics	55.3	64,320,337	0.22	21,520	0.00001000

[* PMPM: Per Member Per Month, ^1^ Per-Device PMPM Contribution = PMPM divided by number of devices. Used as a coefficient to translate device utilization counts into PMPM cost downstream (Table 3)].

**Table 2:** State Non-Group and Employer-Insured Populations, Disability Rate, and Relevant Population.

State	Insured Population		Disability Rate (<65)	Population (<5 & ≥65 Y)	Relevant Population (6–64) ^4^	
	% of Total Population ^1^	Difference from MN (61.6%) ^2^	Difference from MN (7.7%) ^3^	%	%	Number
Alabama	54%	−8%	3.9	24%	76%	4,539,318
Alaska	51%	−11%	1.4	21%	79%	584,655
Arizona	53%	−9%	1.4	25%	75%	5,733,111
California	53%	−8%	1.4	22%	78%	30,775,852
Delaware	54%	−8%	2.0	27%	73%	778,005
Florida	53%	−8%	1.0	27%	73%	17,198,026
Georgia	56%	−6%	1.4	21%	79%	8,906,565
Hawaii	56%	−6%	1.4	27%	73%	1,048,824
Indiana	56%	−6%	2.2	23%	77%	5,362,493
Louisiana	46%	−15%	1.4	24%	76%	3,519,060
Michigan	55%	−6%	2.4	25%	75%	7,646,552
Missouri	57%	−5%	1.4	24%	76%	4,746,800
Nebraska	60%	−2%	0.4	24%	76%	1,543,775
New York	52%	−10%	1.4	24%	76%	15,181,842
North Carolina	53%	−8%	1.6	23%	77%	8,600,039
Oklahoma	50%	−12%	1.4	23%	77%	3,179,055
Rhode Island	55%	−7%	1.4	24%	76%	842,578
Texas	55%	−6%	0.5	20%	80%	25,367,857
Utah	70%	8%	0.3	19%	81%	2,873,590
Vermont	56%	−6%	2.9	26%	74%	475,117
Virginia	57%	−4%	0.6	23%	77%	6,855,443
Washington	58%	−3%	1.4	23%	78%	6,200,791
Wisconsin	60%	−2%	1.4	24.8%	75.%	4,491,536

[*Source: Kaiser Family Foundation (2025); U.S. Census Bureau (2025 population estimates).* ^1^ Insured refers to non-group and employer-sponsored, coverage. ^2^ Insured population difference calculated relative to Minnesota baseline (61.6%). ^3^ Disability rate difference calculated relative to Minnesota baseline (7.7%). ^4^ Relevant population defined as individuals aged 6–64.].

**Table 3:** State Prosthetic and Orthotic Utilization, Costs, and Per Member Per Month (PMPM) Cost Increase.

State	General-Use Prosthetic (GUP), Activity-Specific Prosthetic (ASP)			Orthotics (O)		Variance (V) ^6^	GUP + ASP + O OR ASP + O + V ^7^
	Annual Utilization (GUP) ^1^	Cost (GUP) ^2^	Cost (ASP) ^3^	Annual Utilization (O) ^4^	Cost (O) ^5^	Population PMPM Increase + Disability PMPM Increase	PMPM Increase
Alabama	16,795	0.17	0.05	66,728	0.14	0.03 + 0.01	0.43
Alaska	2163	0.02	0.01	8594	0.02	NA	0.05
Arizona	21,213	0.21	0.06	84,277	0.17	0.04 + NA	0.49
California	113,871	1.14	0.34	452,405	0.93	0.10 + NA	1.37
Delaware	2879	0.03	0.01	11,437	0.02	NA + 0.01	0.04
Florida	63,633	0.64	0.19	252,811	0.52	0.10 + 0.01	1.46
Georgia	32,954	0.33	0.10	130,927	0.27	0.02 + NA	0.39
Hawaii	3881	0.04	0.01	15,418	0.03	NA	0.08
Indiana	19,841	0.20	0.06	78,829	0.16	0.01 + 0.01	0.24
Louisiana	13,021	0.13	0.04	51,730	0.11	0.02 + NA	0.17
Michigan	28,292	0.28	0.08	112,404	0.23	0.03 + 0.01	0.65
Missouri	17,563	0.18	0.05	69,778	0.14	0.01 + NA	0.21
Nebraska	5712	0.06	0.02	22,693	0.05	NA	0.12
New York	56,173	0.56	0.17	223,173	0.46	0.11 + NA	1.31
North Carolina	31,820	0.32	0.10	126,421	0.26	0.06 + 0.01	0.74
Oklahoma	11,763	0.12	0.04	46,732	0.10	0.03 + NA	0.28
Rhode Island	3118	0.03	0.01	12,386	0.03	NA	0.03
Texas	93,861	0.94	0.28	372,907	0.77	0.06 + NA	1.11
Utah	10,632	0.11	0.03	42,242	0.09	NA	0.12
Vermont	1758	0.02	0.01	6984	0.01	NA + 0.01	0.03
Virginia	25,365	0.25	0.08	100,775	0.21	0.01 + NA	0.29
Washington	22,943	0.23	0.07	91,152	0.19	NA	0.26
Wisconsin	16,619	0.17	0.05	66,026	0.14	NA	0.35

[*Source: U.S. Government Accountability Office (2024); Minnesota Department of Commerce (2024); Amputee Coalition; U.S. Census population estimates (2025)*. ^1^ Calculated as Relevant Population × 0.37% (prosthetic utilization rate). ^2^ General-use prosthetic cost calculated using MN Prosthetic per-device cost (0.00001) from Table 1. ^3^ Activity-specific prosthetic cost assumes 30% of users receive additional devices. ^4^ Calculated as relevant population × 1.47% (orthotic utilization rate). ^5^ Orthotic cost calculated using Mn Orthotic per-device cost (0.00000206) from Table 1. ^6^ Variance from either insured population and/or disability prevalence population from MN. NA = variance resulted in less than one cent PMPM. ^7^ PMPM increase reflects combined general-use prosthetic, activity-specific prosthetic, and orthotic costs, or activity-specific prosthetic and orthotic costs only, plus variance PMPM increase, depending on the structure of proposed state legislation.].

**Table 4:** Total Cost, Estimated Savings, and Net Fiscal Impact by State.

State	Savings Through Physical Activity by O&P Users		Cost to All Members		Net Savings to State
	O&P Population (<65) ^1^	Annual Gross Savings (USD) ^2^	Number of Members ^3^	Annual Cost (USD) ^4^	USD ^5^
Alabama	83,523	208,808,634	2,442,153	$12,478,450	$196,330,183
Alaska	10,758	26,894,135	297,589	$163,650	$26,730,485
Arizona	105,489	263,723,112	3,008,883	$17,675,457	$246,047,655
California	566,276	1,415,689,175	16,434,305	$270,883,285	$1,144,805,890
Delaware	14,315	35,788,219	416,233	$210,757	$35,577,462
Florida	316,444	791,109,182	9,148,350	$160,078,740	$631,030,442
Georgia	163,881	409,702,010	4,987,677	$23,256,827	$386,445,182
Hawaii	19,298	48,245,915	584,195	$576,313	$47,669,602
Indiana	98,670	246,674,682	2,997,634	$8,701,915	$237,972,766
Louisiana	64,751	161,876,761	1,625,806	$3,231,303	$158,645,457
Michigan	140,697	351,741,411	4,228,543	$32,797,534	$318,943,877
Missouri	87,341	218,352,779	2,681,942	$6,643,665	$211,709,114
Nebraska	28,405	71,013,631	920,090	$1,336,016	$69,677,615
New York	279,346	698,364,736	7,894,558	$124,006,119	$574,358,618
North Carolina	158,241	395,601,814	4,592,421	$41,006,001	$354,595,813
Oklahoma	58,495	146,238,532	1,589,528	$5,325,185	$140,911,347
Rhode Island	15,503	38,758,582	462,575	$193,546	$38,565,036
Texas	466,769	1,166,921,413	14,003,057	$186,482,515	$980,438,897
Utah	52,874	132,185,142	2,005,766	$2,862,185	$129,322,957
Vermont	8742	21,855,365	266,065	$94,702	$21,760,663
Virginia	126,140	315,350,360	3,935,024	$13,868,218	$301,482,142
Washington	114,095	285,236,363	3,608,860	$11,112,450	$274,123,913
Wisconsin	82,644	206,610,648	2,681,447	$11,328,219	$195,282,428

[*Source: U.S. Census Bureau; Kaiser Family Foundation; Minnesota Department of Commerce (2024).* ^1^ O&P. population aged 6–64 with orthotic and prosthetic needs is calculated as the sum of orthotic utilization and prosthetic utilization from Table 3. ^2^ Calculated as O&P Population × $2500 annual healthcare savings associated with physical activity enabled by orthotic and prosthetic device use. ^3^ Commercial and non-group insured population is calculated by multiplying State Insured Population with Relevant Population. ^4^ Annual cost is calculated as PMPM × 12 × covered population. ^5^ Net savings calculated as estimated annual savings minus annual cost prior to application of sensitivity analysis.].

**Table 5:** Return on Investment (ROI) by Scenario—A Sensitivity Analysis.

State	Annual Cost to All Members	Sensitivity Analysis: Net Savings to State Scenarios			ROI ^1^
	From Table 4 (USD)	Conservative (USD)	Moderate (USD)	High—Impact (USD)	
Alabama	$12,478,450	$8,402,413	$91,925,867	$300,734,500	7.4
Alaska	$163,650	$2,525,764	$13,283,418	$40,177,553	81.2
Arizona	$17,675,457	$8,696,854	$114,186,099	$377,909,211	6.5
California	$270,883,285	−$129,314,367	$436,961,303	$1,852,650,478	1.6
Delaware	$210,757	$3,368,065	$17,683,353	$53,471,572	83.9
Florida	$160,078,740	−$80,967,822	$235,475,851	$1,026,585,033	1.5
Georgia	$23,256,827	$17,713,374	$181,594,177	$591,296,187	7.8
Hawaii	$576,313	$4,248,279	$23,546,645	$71,792,560	40.9
Indiana	$8,701,915	$15,965,553	$114,635,425	$361,310,107	13.2
Louisiana	$3,231,303	$12,956,373	$77,707,077	$239,583,838	24.0
Michigan	$32,797,534	$2,376,607	$143,073,172	$494,814,583	4.4
Missouri	$6,643,665	$15,191,613	$102,532,724	$320,885,503	15.4
Nebraska	$1,336,016	$5,765,347	$34,170,800	$105,184,431	25.6
New York	$124,006,119	−$54,169,645	$225,176,249	$923,540,986	1.8
North Carolina	$41,006,001	−$1,445,819	$156,794,906	$552,396,720	3.8
Oklahoma	$5,325,185	$9,298,468	$67,793,081	$214,029,614	12.7
Rhode Island	$193,546	$3,682,312	$19,185,745	$57,944,327	99.1
Texas	$186,482,515	−$69,790,374	$396,978,191	$1,563,899,604	2.1
Utah	$2,862,185	$10,356,329	$63,230,386	$195,415,528	22.1
Vermont	$94,702	$2,090,834	$10,832,980	$32,688,345	114.4
Virginia	$13,868,218	$17,666,818	$143,806,962	$459,157,322	10.4
Washington	$11,112,450	$17,411,186	$131,505,731	$416,742,094	11.8
Wisconsin	$11,328,219	$9,332,845	$91,977,104	$298,587,752	8.1

[Source: Modeled estimates using Minnesota Department of Commerce actuarial data, U.S. Census Bureau population data, and Kaiser Family Foundation insurance coverage estimates. ^1^ ROI calculated as Net Savings to state Scenarios divided by Annual Cost Annual Cost to all members. Values greater than 1.0 indicate net positive fiscal return.].

## Data Availability

All data used in this analysis were obtained from publicly available sources, including the Minnesota Department of Commerce, U.S. Census Bureau, Kaiser Family Foundation, American Heart Association, and U.S. Government Accountability Office publications. No proprietary patient-level datasets were utilized. Calculations were conducted using publicly derived state-level population, utilization, and cost estimates described throughout the Section 2.
